# Systematic review of the quality of care provided to sick children in Ethiopian health facilities

**DOI:** 10.7189/jogh.14.04243

**Published:** 2024-11-01

**Authors:** Negalign Berhanu Bayou, Biruk Hailu Tesfaye, Kassahun Alemu, Alemayehu Worku, Lisanu Tadesse, Delayehu Bekele, Getachew Tolera, Grace Chan, Tsinuel Girma Nigatu

**Affiliations:** 1 Health System and Reproductive Health Research Directorate, Ethiopian Public Health Institute, Addis Ababa, Ethiopia; 2 Department of Health Policy and Management, Institute of Health, Jimma University, Jimma, Ethiopia; 3 Maternal, Child and Adolescent Health lead Executive Office, Ministry of Health, Addis Ababa, Ethiopia; 4 HaSET Maternal and Child Health Research Program, Addis Ababa, Ethiopia; 5 Schools of Public Health, College of Health Sciences, Addis Ababa University, Ethiopia; 6 Department of Gynecology and Obstetrics, St. Paul’s Hospital Millennium Medical College, Addis Ababa, Ethiopia; 7 Research and Technology Transfer, Ethiopian Public Health Institute, Addis Ababa, Ethiopia; 8 Department of Pediatrics, Boston Children's Hospital, Harvard Medical School, Boston, Massachusetts, USA; 9 Department of Epidemiology, Harvard University T H Chan School of Public Health, Boston, Massachusetts, USA; 10 The University of British Columbia, Addis Ababa, Ethiopia

## Abstract

**Background:**

Despite the increasing number of primary studies on the quality of health care for sick children in Ethiopia, the findings have not been systematically synthesised to inform quality improvement in policies or strategies. This systematic review provides a narrative synthesis of published evidence on the quality of care provided to sick children in Ethiopia's health facilities and on related barriers and enablers.

**Methods:**

We searched studies that measured the structure, process, and outcome measures of quality of care as proposed by Donabedian’s framework. We searched in PubMed/Medline, EMBASE, and Web of Science using the Population, Concept, and Context (PCC) framework. Grey literature was searched in Google Scholar and institutional websites. We appraised the studies’ quality using the Mixed Method Quality Appraisal Tool version 2018. Data were analysed using content thematic analysis and presented using a narrative approach.

**Results:**

We included 36 of 701 studies. Thirty (83.3%) were nonexperimental including 21 (70%) cross-sectional studies and five (16.7%) qualitative studies. Of the 31 facility-based studies, 29 (93.5%) were conducted in public facilities. The structural, technical, and interpersonal processes of care were low quality. While some studies reported the effectiveness of interventions in reducing child mortality, the uptake of services and providers’ and caretakers’ experiences were suboptimal. The major structural barriers to providing quality care included inadequacy of essential drugs, supplies and equipment, training, clinical guidelines, and ambulance services. Caretakers’ non-compliance to referral advice was a common demand-side barrier. The enabling factors were implementing various health system strengthening interventions including quality improvement strategies such as user-centred service delivery and optimising engagement of community-level structures such as health promotors and religious leaders to create demand.

**Conclusions:**

The quality of care provided to sick children in health facilities is generally low in Ethiopia. Shortages of essential drugs, supplies and equipment, physical space, water, and electricity; and human resource-related challenges such as shortage, training, supervision, and retention were common structural barriers. Various health systems strengthening and quality improvement interventions, ranging from enhanced demand creation to realising a reliable and consumer-centred service delivery were key enablers. More research is needed on the quality of care provided in private facilities.

**Registration:**

PROSPERO: CRD42021285064.

Ethiopia has reduced the child mortality rate from 87 to 55 deaths per 1000 live births between 2009–2019. However, the reduction in neonatal mortality rate is not significant compared to infant and child mortality rates; 44% of under-5 deaths were neonatal deaths [1]. Poor-quality services have been shown to be risk factors for child mortality in high burden countries despite substantial increases in access to essential health services during the past two decades [2].

The quality of child health care remains a concern in Ethiopia despite the relatively increasing coverage of effective interventions and increasing interest in understanding the quality of care provided to sick children [3]. Improving both the uptake and quality of primary childcare services is imperative for further reduction of under-5 mortality rates [4].

Donabedian’s structure-processes-outcomes framework is the most commonly used tool for health care quality assessment [5] with the assumption that better health care produces better health outcomes [6]. Several studies ranging from small scale to nationwide and from community based to facility-based, have measured one or more of the components of quality of childcare services in Ethiopia [7-9].

Despite the growing number of primary studies on the quality of care for sick children, individual studies cannot inform whether the results apply across specific settings. To the best of our knowledge, no previous work has attempted to systematically identify and synthesise such evidence in Ethiopia. The evidence would help policymakers, practitioners, and researchers to address the identified gaps in the quality of care. Therefore, this systematic review (SR) aimed to determine the quality of care provided to sick children in Ethiopia's health facilities, and the barriers and enabling factors related to quality care provision.

## METHODS

The Preferred Reporting Items for Systematic Review and Meta-Analysis Protocols (PRISMA-P) 2015 statement was used to write the protocol [10]. This SR protocol was registered on the International Prospective Register of Systematic Reviews (PROSPERO) # CRD42021285064.

### Population and outcomes of interest

We determined the eligibility of the review question using the Population, Concept and Context (PCC) framework suggested by the Joanna Briggs Institute [11]. The primary population of interest included sick under-5 children, their caretakers, service providers and managers. The concept referred to the quality of care provided to sick under-5 children as defined by Donabedian’s structure-process-outcome framework.

Structure was assessed in the context of service delivery, including drugs, supplies, equipment, human resources, and organisational characteristics. Process was assessed as a set of actions and services which made up the actual care provided to the sick child, including interpersonal and clinical actions. Outcome or clinical outcomes included death or cure and complication, and quality of care as perceived by caretakers, providers, and managers. Service uptake or coverage was also considered as an intermediate outcome. Together with the other outcomes, measures of key actors’ experiences would serve as proxies for a comprehensive understanding of health system quality, not just clinical or service quality. Thus, responsiveness measures, e.g. trust and confidence in health system, were captured whenever reported. Finally, as applied to this review, the context domain of the PCC framework pertains to those studies conducted in Ethiopia.

### Eligibility criteria

Studies that satisfied the following inclusion criteria were: qualitative, quantitative (i.e. randomised, non-randomised and descriptive studies), or mixed methods; conducted in Ethiopia; reported evidence of quality focused on at least one component of Donabedian’s structure-process-outcome framework; included sick under-5 children or their primary caretakers, service providers and managers as the populations of interest; published in peer-reviewed and grey literature (i.e. theses and dissertations); reported in English; and published until 23 February 2022. We excluded reports on the quality of preventive and promotive services provided, e.g. vaccination, growth monitoring, and vitamin A supplementation, reports on the quality of home-based care, e.g. breastfeeding, more fluids, and feeding, and review articles.

### Search strategy

We thoroughly searched for relevant studies in PubMed/Medline, EMBASE and Web of Science databases. Grey literature was also searched from Google Scholar, university repositories and government websites. Reference lists of eligible studies were also explored. Keywords were combined using Boolean terms ‘AND’ and ‘OR’ to search for eligible studies: ‘child’ OR ‘children’ OR ‘under 5’ OR ‘under-5’ OR ‘under five’ OR ‘under-five’ OR ‘infant’ OR ‘infants’ OR ‘neonate’ OR ‘neonates’ OR ‘newborn’ or ‘newborns’ OR ‘baby’ OR ‘babies’ AND ‘sick’ OR ‘illness’ OR ‘disease’ OR ‘diseases’ AND ‘mother’ OR ‘mothers’ OR ‘caregiver’ OR ‘caregivers’ OR ‘caretaker’ OR ‘caretakers’ OR ‘care takers’ AND ‘quality’ AND ‘child health care’ OR child health care OR ‘newborn care’ OR ‘pediatric care’ OR ‘paediatric care’ OR ‘management of childhood illness’ OR ‘integrated management of childhood illness’ OR IMCI OR ‘community case management’ OR CCM OR ‘integrated community case management’ OR iCCM OR ‘community-based newborn care’ OR CBNC AND Ethiopia. A detailed search strategy is provided in Table S1 in the **Online Supplementary Document**.

### Study selection

A three-step process, adapted from the 2020 PRISMA approach [12], was followed to select studies including identification, initial screening of abstracts, and final eligibility assessment after full-text screening. Two review authors (NBB and BHT) removed duplicate records and screened the title of the studies from the electronic databases. We created an EndNote X9 library to import the identified studies, remove the duplicates, and archive the eligible articles. The abstracts of these identified studies were independently scanned to exclude ineligible studies. Finally, full-length articles were reviewed using the inclusion criteria. The 2020 PRISMA framework was adapted to present the screening results of the review to include search date, type of database, keywords, and numbers of retrieved and eligible studies. Discrepancies between the two review authors (NBB, and BHT) were resolved in discussion with other co-authors.

### Data abstraction and management

We extracted data using a data charting form. Two review authors (NBB and BHT) independently extracted author and year of publication, study title, objective/aim, design, population(s), setting (i.e. health facility (type and level) vs community-based), sample size and sampling technique, data collection method(s), type(s) of disease-focused, types of intervention or measure of quality measured (structure, process and/or outcome) and key findings on quality-of-care measures and conclusions. Discrepancies between the reviewers were resolved using a consensus method.

### Collating, summarising, and reporting the results

We conducted a mixed studies review with a qualitative evidence synthesis approach using a databased convergent thematic synthesis [13–15]. We identified themes from qualitative studies or qualitative aspects of mixed methods studies, while variables used in quantitative studies or quantitative aspects of mixed methods studies were transformed into qualitative codes. A databased convergent synthesis design, rather than a mixed synthesis process, was chosen aimed at corroborating results from qualitative and quantitative methods. All the extracted data (i.e. both qualitative and quantitative) were analysed using the same thematic synthesis method. Categories or themes were then developed using the predefined Donabedian’s structure-process-outcome components of quality measurement. Results of the synthesis were presented using tables and narrative and descriptive summaries. The results were reported using PRISMA 2020 statement guideline [12].

### Risk of bias and quality assessment

Two independent reviewers (NBB and BHT) evaluated the quality of the screened studies reviewed using the Mixed Method Quality Appraisal Tool (MMAT) version 2018 (http://mixedmethodsappraisaltoolpublic.pbworks.com). The tool provides a more efficient appraisal by limiting to core criteria. It focuses on five core methodological quality criteria for five study designs: qualitative, randomised controlled trial, non-randomised, quantitative descriptive and mixed methods. Overall, the tool includes 25 criteria and two screening questions to assess relevance, design, adequacy and methodology, data collection and analysis, and results. The review authors (NBB and BHT) graded the quality of each study by calculating the total percentage quality score. A paper was classified as high quality if it scored 76% and above; of average quality, if it scored between 51 and 75%; and of low quality if it scored ≤50%. Accordingly, 35 studies scored ≥80% and were judged to be of high quality, one study scored 60% and was judged to be of average quality, and no study was judged to be of low quality. Inconsistencies between the reviewers were resolved by discussion with other review co-authors.

## RESULTS

**Figure 1:**
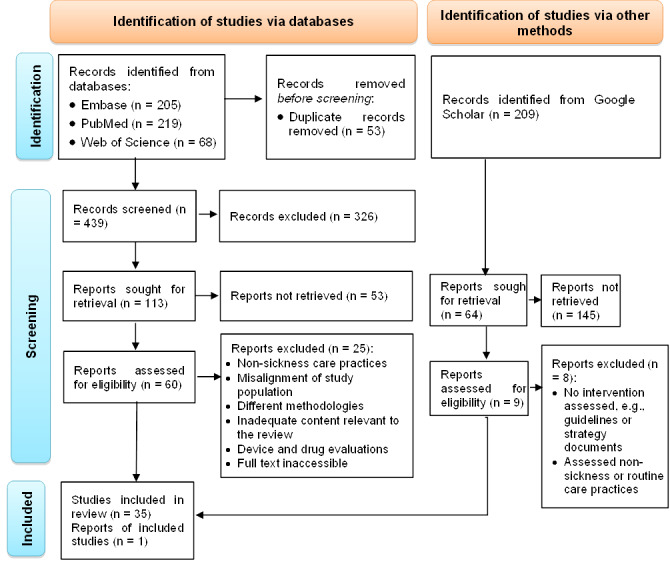
PRISMA 2020 flow diagram of the articles searched and selected for the systematic review of quality of health care provided to sick children in health care facilities in Ethiopia, 2022.

We identified a total of 701 studies, of which 53 were duplicates. After removing the duplicates, we conducted initial title and abstract screening for 648 studies. We retrieved a total of 177 studies and excluded 471 using the eligibility criteria for full-text screening. With further screening, we retained 69 studies for full-text review, of which a total of 36 were included in the final review. Reasons for exclusion after full article screening included reporting non-sickness or routine care practices, misalignment of study populations, different methodologies, inadequate content relevant to the review (e.g. reporting user-related factors for service utilisation), reporting on a specific device or drug evaluations, and full-text inaccessibility (**Figure 1**).

### Characteristics of the included studies

Four (11.1%) of the 36 included studies were randomised controlled trials and two (5.6%) were non-randomised pre-post interventional studies. The remaining 30 studies (83.3%) employed a nonexperimental design. The majority of these, 21 (70%), were cross-sectional surveys and five (16.7%) were qualitative studies, while one (3.3%) was a time series. The study population varied from health facilities in nine (25%) studies which involved only resource inventory and review of clinical records in two studies (5%), to individuals (i.e. children, caretakers, health workers and facility managers) in 25 studies (70%). Six studies (17%) used caretakers as their principal study population, followed by health workers and facility managers used in four studies (11%). Sixteen studies (44%) exclusively focused on various newborn illnesses, while the remainder (56%) focused on one or more common childhood illnesses, including diarrhoea, malaria/fever, pneumonia, and severe acute malnutrition (SAM) (Table S2 in the **Online Supplementary Document**).

Of the studies, 26 (72.2%), were health facility-based [7,9,16–38], five (18.9%) were community-based [39–43], and five (18.9%) were conducted in both settings [44-48]. Of the facility-based studies, including those conducted in both settings (n = 31), the majority, 29 (93.5%), involved public health facilities [7,9,16–23,25–38,44–48]. The remaining two studies (6.5%) were conducted in both public and private facilities [8,24] (Table S3 in the **Online Supplementary Document**).

### Structure

Out of the 36 studies reviewed, 19 (52.8%) reported structural measures of quality of care provided to sick children either in a stand-alone study or in combination with process and/or outcome measures [7,8,16,18,22,24–27,29–32,34,37–39,46–48]. Only two of these studies that applied the pathway to child survival analysis [46], and that identified gaps in the provision of quality integrated community case management (iCCM) services provided by health extension workers (HEWs) and caregivers’ adherence to prescribed medicines [32], reported mixed findings, i.e. both adequacy and inadequacy of the quality of the structural measures. For instance, according to a cross-sectional study that assessed the strength of iCCM implementation and quality of care provided by HEWs [31], Most HEWs (78.8%) were trained in iCCM, and 87% received supervision three months prior to the study. They also showed that 70% of health posts had all essential commodities for iCCM. Similarly, Sintayehu, et al. [41], revealed the availability of neonatal resuscitation corners (97.4%) and essential equipment for newborn resuscitation (85%) in the health facilities. However, the availability of newborn resuscitation guidelines was 63%, and only 25.1% of health professionals had ever received refresher training on neonatal resuscitation [41].

In the remainder of the studies [7,8,16,18,23–27,29,30,32,37–39,45–48], the structural quality was reported to be substandard, regardless of how the specific measures were measured through resource inventory, or from providers’ or users’ perspectives. Likewise, the level of care at the health post (HP), health centre (HC) or hospital level was substandard. The overall structural standards for a neonatal intensive care unit (NICU) were met by 63% of the studied primary hospitals [16], and 65.2% of the required equipment, and 72.2% of medicines, were met in the NICU ward of a referral hospital [30]. Similarly, Biadgo, et al. [7] reported that only 15.6% of the studied health facilities fulfilled the standards for quality childcare provision. The stockout of essential drugs, supplies and equipment at HP level has also been cited in other studies [29,32,41,48].

Three studies [8,27,29] have identified gaps in the availability of skilled staff. Getachew, et al. [8] and Jebessa, et al. [27] reported that about half of service providers received sick childcare training in the two years preceding the study, while Ketaro, et al. [29] showed that almost all the providers did not receive any similar training during the same reference period. The latter study also reported a lack of a written job description as a work aid for service providers.

### Process

Twenty-three studies (63.9%) have assessed the process measures of quality of care provided to sick children [7,8,11,17,18,21–35,39–41,45,46]. A relatively higher level of adherence of providers to the recommended standards of care was reported by three (13%) of these studies [32,39,40]. For example, an evaluative study that examined the effectiveness of conducting community case management of malaria among children in urban settings [39] showed that almost all (99%) of children with fever were prescribed the correct dose of Artemisinin-based combination therapy (ACT) for their age by community medicine distributors. Health extension workers’ knowledge of assessment, classification, and referral of children with severe illness was also reported to be above 80% by Najjemba, et al. [32].

Approximately two-thirds (60.9%) of the studies reported that both the clinical and interpersonal aspects of care provided to sick children were generally of low quality [7–9,18,24,25,27,29,31,32,34,35,39,46]. The gap ranged from no child being correctly assessed [18] to an overall compliance of 68% [25]. A study that examined the know-do gap of providers revealed low performance with an average score of 34%. The gap between knowledge and performance was large for treatment and counselling items (39%), and among doctors [24]. Sintayehu et al. [34] also demonstrated poor retention of basic neonatal resuscitation skills by midwives and nurses (11.2%). Gebremedhin et al. [25] similarly revealed that very severe diseases were not treated according to the national CBNC programme implementation guideline, and identification of neonatal sepsis cases was poor. Getachew et al. [8] also identified that respiratory rate was counted in 56% of children, their temperature was checked in 77% of cases of suspected pneumonia, and dehydration was assessed in 54% of children who had diarrhoea with dehydration. Degefie et al. [22] cited that HEWs could identify only 57% of sick newborns with at least one sign of possible severe bacterial infections (PSBI) to provide antibiotic treatment when referral to higher levels was not possible. Daka et al. [9], Mash et al. [46], Miller et al. [31], and Tamiru et al. [35] have also found gaps in HEWs’ performance on iCCM implementation.

Out of 20 studies that reported on interpersonal communication between providers and caretakers, five (21.7%) identified gaps [8,27,29,31,46]. In a cross-sectional study that assessed the quality of integrated management of childhood illness (IMNCI) services in health centres of Jimma zone, Southwest Ethiopia [29], most providers reported little or no communication with caretakers at discharge. Other studies found that none [29] and only 2% [8] of the providers used a visual aid during consultations. From a caretakers’ perspective, providers advised nearly half (48.4%) on follow-up care according to Ketaro et al. [29]. However, this was only 13% in another study [46], which also reported that a quarter of the caretakers understood treatment advice provided.

### Outcome

The majority, 26 (72.2%), of the reviewed studies measured one or more of the outcome measures of quality [8,16,17,19,20,22,23,25–33,38–44,46–48]. Ten (38.5%) of these studies investigated coverage or utilisation of child health interventions [19,20,22,31,40,41,43,46–48], followed by nine (34.6%) on the perceived quality of care both from caretakers’ and providers’ sides. Eight of the latter group of studies (88.9%) explored caretakers’ views [8,23–25,29,30,33,39], while two (22.2%) explored providers’ views [27,44]. In addition, seven (26.9%) studies assessed health outcomes at both individual and population levels. Five of the studies (71.4%) looked at the effectiveness of interventions in reducing childhood mortality at a population level [16,22,26,31,42], while four (57.1%) examined clinical or treatment outcomes, including recovery and complication rates and adverse effects of clinical treatment [16,19,26,28].

Studies generally found that the coverage or utilisation of community-based child survival interventions was suboptimal in Ethiopia [19,20,22,31,40,41,43,48]. For example, the uptake of life-saving interventions ranged from 9.3% for iCCM services [41] to 77.7% for the treatment of PSBI [20]. The perceived quality of care was similarly reported to be low, mainly towards services provided at a community level. For example, mothers believed that distance from home to the nearest HP was not a barrier for them if the quality of services provided by HEWs was perceived as good. They lacked trust in the HEWs’ ability to treat children at home [44]. Getachew et al. [8] also reported that caretakers preferred private providers over HP-level care. Jebessa et al. [27] similarly revealed a compromised quality of care provided to sick newborns from mothers’, health care providers’ and facility administrators’ views. According to Ketaro et al. [29], 23.4% of caretakers were dissatisfied with waiting times and 33.6% were dissatisfied with consultations or treatment given by providers.

With regards to the individual and population level health outcomes, four studies [20,22,26,42] reported the effectiveness of the investigated services in reducing, or in their potential to reduce, child mortality. For example, studies by Berhane et al. [20] and Degefie et al. [22] have shown that infants with possibly severe bacterial infection (PSBI) can effectively be managed as outpatients at HP or HC level when referral to a hospital is not feasible. A slight decrease in perinatal mortality was also observed in a time series analysis of the impacts of quality improvement intervention on maternal and newborn health in four Ethiopian regions [26]. Tadesse et al. [42] also revealed a 17.7% reduction in childhood diarrhea with the model household creation strategy of health extension programme (HEP). In contrast, few studies reported unfavorable health outcomes [28,40]. A study [40] found an insignificant contribution of the iCCM programme to child mortality reduction. Kabalo and Seifu [28] similarly reported a lower recovery rate (64.9%) of children with severe acute malnutrition (SAM) treated using the outpatient therapeutic feeding programme.

### Barriers

Nine studies [25%] identified supply-side barriers to quality care provision [18,21,24,27,36–38,41,47], while three studies [8.3%] reported demand-side barriers [21,22,27]. Barriers related to adequacy of infrastructure, human resources and perceived poor quality of care were included in the frequently cited supply-side barriers. Shortages of essential drugs, supplies and equipment, physical space, water, and electricity were noted as infrastructural bottlenecks for quality care provision [18,24,27,36–38,41,47]. Human resource-related challenges such as shortage, training, supervision, and retention were reported in five studies [18,21,27,37,47]. The lack of clinical guidelines in health facilities was a related gap emphasised in two studies [21,27]. Three studies [21,27,36] identified the lack of ambulance service as a barrier to the functionality of the referral system, and a lack of funds was reported by another study [37].

Perceived poor quality of care provided by health facilities has been cited in four studies as a barrier to service uptake [18,21,41,47]. The specific gaps included facility closure and inconvenient opening hours [36,42], long consultation time [18], under-resourced physical facilities and lack of confidence in HEWs’ skills [47], and services provided by referral facilities [21].

From the demand side, caretakers’ non-compliance to referral advice and economic insecurity were reported as barriers [21,22,27]. For example, Degefie et al. [22] reported that 90% of caretakers of infants with PSBI refused referral. According to Jebessa et al. [27], users are less likely to accept interventions if they perceive that there would be unaffordable financial costs.

### Enablers

The reviewed studies suggested solutions pertinent to the barriers discussed above. Various health systems strengthening, and quality improvement interventions, have been recommended by many studies, ranging from enhanced demand creation to realising a reliable and consumer-centered service delivery [8,21,23,26,29,33,37,39,40,43,44,48].

A significant effect of infrastructural capacity improvement on health workers’ adherence to recommended care practices has been established by Hagaman et al. [27]. Abayneh et al. [17] also found that implementing quality improvement interventions significantly improved health workers’ performance of IMCI. Maintaining a consistent supply of essential drugs, supplies and equipment [29,47], and ensuring a functional referral system [21] were among the specific solutions suggested to realise quality service delivery to improving health outcomes.

Some studies [8,23,40,44] also emphasised the critical role of building the capacity of the health workforce through training, supervision, and availing clinical guidelines and protocols. For instance, iCCM-trained workers provided good quality care [40], and the training of providers also resulted in higher client satisfaction [8].

Finally, four studies pointed out enabling factors for demand creation [19,21,43,47]. Usman et al. [37] recommended engaging community leaders such as HDA and religious leaders, as well as model families to maximise their role as information disseminators and influencers to resonate their best practices. Mengistu et al. [47] cited establishing and making use of community feedback mechanisms to enhance community engagement and respond to their health needs in a timely manner. In another study, families believed that receiving training on model family packages of HEP was significantly associated with the uptake of iCCM services [43]. Improving caretakers’ awareness of signs of illness was also recommended to improve their compliance to referral advice [21].

## DISCUSSION

The results showed that the quality of care provided to sick children primarily in public health facilities was generally low in Ethiopia in terms of all the quality components. Shortage of essential drugs, supplies, equipment training, clinical guidelines and ambulance services were the common supply-side barriers, while low service uptake and caretakers’ non-compliance to referral advice were the demand-side barriers. For those studies conducted during and after 2019, COVID-19 pandemic might have contributed to the observed under achievements due to shifting health care resource towards the pandemic response, and limited access to services due to lockdown measures.

Overall, our findings are consistent with the existing literature. For example, an analysis of facility surveys in nine countries revealed the failure of primary care facilities to carry out their function with a mean quality score of 0.41 (out of 1), an index for evidence-based care, competent systems, and user experience domains. Ethiopia scored the lowest (0.32) in this study, while Namibia scored the highest (0.46) [49]. As noted in Rockers et al. [50], ensuring high clinical quality, along with client-centred service organisation and delivery, increased uptake and ensuring better health outcomes increases users’ trust in the health system. This would, in turn, lead to increased service uptake and better health outcomes. It is also evident that poor-quality care is responsible for 60% of deaths due to treatable conditions, while the remainder are due to the non-use of health services [49]. Thus, ensuring high-quality care would prevent one million newborn deaths annually [49].

In terms of structural measures of care quality, this review showed low readiness of health facilities to provide sick childcare services at all health facility levels. A similar finding has been reported in low-and-middle income countries (LMICs) where health facilities were often underequipped and understaffed [49]. The authors attributed poor-quality care to gaps in the knowledge, skills, and motivation of health workers. Although structural measures of care quality are weakly related to the content of care delivered [51], high-quality care cannot be expected in such constrained settings [6]. To benefit from the implementation of effective interventions, the health system should ensure that health facilities, health professionals, essential medicines and equipment are always available to meet the required standards of care [52]. The observed widespread nature of the gaps across settings and disease types may suggest that structural barriers are system-wide issues.

Consistent with several previous studies, our review also revealed that both the clinical and interpersonal processes of care provided to sick children were generally of low quality. Leslie et al. [51] analysed nationally representative health systems surveys to see the association between in-service training and supervision, and the quality of sick childcare in seven sub-Saharan Africa (SSA) countries. Results demonstrated a poor quality of care with health workers complying with less than half of the recommended practices. Other studies also reported non-compliance of providers to the recommended clinical practices with frequent misdiagnoses of deadly conditions such as pneumonia [49,53–56], diarrhoea [53,56] and newborn asphyxia [49].

The suboptimal process quality may be partly explained by the shortage of required inputs such as drugs, supplies, equipment, and laboratory facilities, even when health workers know the importance of these critical resources in creating an enabling care setting [57]. Quality of care processes determines both service uptake and health outcomes. Implementation of interventions, such as IMCI or community case management (CMM) is not a guarantee for improved health outcomes, as the quality of care is key to ensuring adequate case management, encouraging the use of the services and attaining health outcomes [58]. The observed gaps in performance across studies suggest the quality improvement potential in the country. Non-compliance to evidence-based care practices would lead to adverse conditions such as treatment delays, disease progression, waste of resources, catastrophic out-of-pocket expenditures, and eventually death [49,59].

Poor-quality care also includes the underuse of available effective services [53]. The suboptimal coverage or utilisation and providers’ and caretakers’ experiences with the child survival interventions have also been cited previously. For example, Kruk et al. [49] noted that an average of 34% of people in LMICs had poor user experience due to lack of attention or respect (41%), long waiting times (37%), poor communication (21%), or short service time (37%). The study also cited that only 43% of providers informed caregivers about their child’s diagnosis. The finding can partly be explained by the inadequate quality of structural and process components observed in the review [6,51].

Perceived poor quality of care can affect health care utilisation patterns and bypass of facilities [60–62]. Although satisfaction can be affected by user-related factors such as sociodemographic, health conditions, care experiences, expectations, and courtesy bias [63], it is related to objective measures of process quality and health outcomes [64]. As a result, the validity of this measure of quality may be challenged. For instance, illiterate or less experienced users mostly show high satisfaction for apparently poor-quality services. In contrast, expecting but not receiving services that are not designated, e.g. antibiotics for common cold, may dissatisfy users [49].

The interventions investigated by some of the reviewed studies were reported to be effective in reducing child mortality. However, good health outcomes are not always attributable to good procedures due to multiple factors which, in addition to the treatment protocol, determine health outcomes [49,65]. Since quality of care is a sensitive indicator of a well-functioning health system [49], the findings on all the three quality domains highlight that the health system would effectively improve child survival by improving both quality and coverage of health care in the country.

We also identified the inadequacy of essential drug supplies and equipment, staff training, clinical guidelines, physical space, water, electricity, and ambulance services as supply side barriers. Such structural barriers impact not only service utilisation, but also on the effectiveness of interventions and client perceptions [58]. Perceived poor quality of care was also a blockade for service uptake. Caretakers’ non-compliance to referral advice, due to the associated costs and unfavourable illness perceptions of caretakers, were the demand side barriers. These findings endorse the low quality of care both for technical and interpersonal attributes of process quality. Enhancing health care system capacity through ensuring an enabling service delivery setting and developing providers’ capacity is essential.

Communities with high levels of social capital, where material and psychosocial resources are shared by members who work together to address their problems collectively, are more likely to address their problems [66]. Implementing various health systems strengthening and quality improvement interventions, such as enhanced demand creation and consumer centred service delivery were the enablers identified in this review. Engaging community leaders such as the health development army (HDA), model families and religious leaders, and using community feedback mechanisms, enabled demand creation. The findings highlight the need to tap the existing community-level structures as a vehicle for demand creation. For instance, enhancing community conversations and social support systems, can then help improve caretakers’ compliance with referral advice.

This SR had strengths and weaknesses. Being the first synthesis report of the available evidence on the quality of care provided to sick children in the country, the work has generated wide-ranging evidence to inform policy and practice decisions and areas for future research. The scope of the review was broad, which we believe provided a comprehensive review of care quality, all derived from studies of good quality. However, the linearity assumption of the Donabedian’s quality assessment framework [6] may mask the complex interactions that exist among quality components, and between components and the broader environment. Efforts were made to understand the quality of care for sick children from a systems perspective by extending Donabedian’s framework, which primarily intends to measure clinical care quality [6], to include population-level metrics such as service coverage. However, the review did not synthesise evidence on the non-health effects of various child survival interventions such as economic values (e.g. cost-effectiveness, cost-benefit, and catastrophic expenditures due to poor quality care) and financial risk protection mechanisms. Such information would enable a better understanding of how the health system operates toward reducing child mortality.

## CONCLUSIONS

The quality of care provided to sick children in health facilities was generally low in Ethiopia. The readiness of health facilities to provide childcare services to sick children was low at all levels. Both technical and interpersonal processes of care were also of low quality. Mixed findings were observed on outcome quality. Some studies reported the effectiveness of interventions in reducing child mortality, while uptake of child survival interventions and providers’ and caretakers’ experiences are suboptimal outcomes. The major structural barriers to providing quality care included inadequacy of essential drugs, supplies and equipment, training, clinical guidelines, and ambulance services. Caretakers’ non-compliance to referral advice was a demand-side barrier. Implementing various health systems strengthening and quality improvement interventions, such as consumer-centred service delivery and enhanced demand creation were the enabling factors identified.

## Additional material


Online Supplementary Document
