# Supplementary material for: Systematic review of the quality of care provided to sick children in Ethiopian health facilities
Source: J Glob Health. 2024 Nov 1;14:04243. doi: 10.7189/jogh.14.04243 (PMC11525845; doi:10.7189/jogh.14.04243)
Supplement: Online Supplementary Document [file jogh-14-04243-s001.pdf]

Online Supplementary Document

Table 1: Detailed search strategy by database, a systematic review of quality of healthcare provided to sick children in healthcare facilities in Ethiopia, 2022

Search	Search detail	Number	Date
PubMed			
#1	**child**:ti,ab,kw OR **children**:ti,ab,kw OR **'under 5'**:ti,ab,kw OR **'under five'**:ti,ab,kw OR **infant**:ti,ab,kw OR **infants**:ti,ab,kw OR **neonate**:ti,ab,kw OR **neonates**:ti,ab,kw OR **newborn**:ti,ab,kw OR **newborns**:ti,ab,kw OR **baby**:ti,ab,kw OR **babies**:ti,ab,kw OR **'baby'**/exp	2,496,458	23-Feb-22
#2	**sick**:ti,ab,kw OR **illness**:ti,ab,kw OR **disease**:ti,ab,kw OR **diseases**:ti,ab,kw OR **'illness'**/exp	27,429,466	23-Feb-22
#3	**mother**:ti,ab,kw OR **mothers**:ti,ab,kw OR **caretaker**:ti,ab,kw OR **caretakers**:ti,ab,kw OR **'care taker'**:ti,ab,kw OR **'care takers'**:ti,ab,kw OR **caregiver**:ti,ab,kw OR **caregivers**:ti,ab,kw OR **'care giver'**:ti,ab,kw OR **'care givers'**:ti,ab,kw	425,532	23-Feb-22
#4	**quality**:ti,ab,kw OR **'quality'**/exp	1,693,454	23-Feb-22
#5	**'child health care'**:ti,ab,kw OR **'child healthcare'**:ti,ab,kw OR **'newborn care'**:ti,ab,kw OR **'pediatric care'**:ti,ab,kw OR **'paediatric care'**:ti,ab,kw OR **'management of childhood illness'**:ti,ab,kw OR **'health care'**/exp OR **'newborn care'**/exp OR **'paediatric care'**/exp OR **'pediatric care'**/exp OR **imci**:ti,ab,kw OR **'integrated management of childhood illness'**:ti,ab,kw OR **'community case management'**:ti,ab,kw OR **'community case management'**/exp OR **ccm**:ti,ab,kw OR **'integrated community case management'**:ti,ab,kw OR **iccm**:ti,ab,kw OR **'community-based newborn care'**:ti,ab,kw OR **'community based newborn care'**:ti,ab,kw OR **cbnc**:ti,ab,kw	6,135,563	23-Feb-22
#6	**ethiopia** OR **'ethiopia'**/exp	33,998	23-Feb-22
#7	#1 AND #2 AND #3 AND #4 AND #5 AND #6	2,895	23-Feb-22
#8	#7 AND Abstract, Classical Article, Clinical Trial, Electronic Supplementary Materials, Evaluation Study, Multicenter Study, Observational Study, English	214	
Embase			
#1	child:ti,ab,kw OR children:ti,ab,kw OR 'under 5':ti,ab,kw OR 'under five':ti,ab,kw OR infant:ti,ab,kw OR infants:ti,ab,kw OR neonate:ti,ab,kw OR neonates:ti,ab,kw OR newborn:ti,ab,kw OR newborns:ti,ab,kw OR baby:ti,ab,kw OR babies:ti,ab,kw OR 'baby'/exp	2496458	23-Feb-22
#2	sick:ti,ab,kw OR illness:ti,ab,kw OR disease:ti,ab,kw OR diseases:ti,ab,kw OR 'illness'/exp	27429466	23-Feb-22
#3	mother:ti,ab,kw OR mothers:ti,ab,kw OR caretaker:ti,ab,kw OR caretakers:ti,ab,kw OR 'care taker':ti,ab,kw OR 'care takers':ti,ab,kw OR caregiver:ti,ab,kw OR caregivers:ti,ab,kw OR 'care giver':ti,ab,kw OR 'care givers':ti,ab,kw	425532	23-Feb-22
#4	quality:ti,ab,kw OR 'quality'/exp	1693454	23-Feb-22
#5	'child health care':ti,ab,kw OR 'child healthcare':ti,ab,kw OR 'newborn care':ti,ab,kw OR 'pediatric care':ti,ab,kw OR 'paediatric care':ti,ab,kw OR 'management of childhood illness':ti,ab,kw OR 'health care'/exp OR 'newborn care'/exp OR 'paediatric care'/exp OR 'pediatric care'/exp OR imci:ti,ab,kw OR 'integrated management of childhood illness':ti,ab,kw OR 'community case management':ti,ab,kw OR 'community case management'/exp OR ccm:ti,ab,kw OR 'integrated community case management':ti,ab,kw OR iccm:ti,ab,kw OR 'community-based newborn care':ti,ab,kw OR 'community based newborn care':ti,ab,kw OR cbnc:ti,ab,kw	6135563	23-Feb-22
#6	ethiopia OR 'ethiopia'/exp	33998	23-Feb-22
#7	#1 AND #2 AND #3 AND #4 AND #5 AND #6	100	23-Feb-22

Web of Science			
#1	Child OR children OR 'under 5' OR 'under-5' OR 'under five' OR 'under-five' OR infant OR infants OR neonate OR neonates OR newborn or newborns OR baby OR babies (All Fields) and sick OR illness OR disease OR diseases (All Fields)	917,191	23-Feb-22
#2	Child (All Fields) or children (All Fields) or under 5 (All Fields) or under-5 (All Fields) or under five (All Fields) or under-five (All Fields) or infant (All Fields) or infants (All Fields) or neonate (All Fields) or neonates (All Fields) or newborn (All Fields) or newborns (All Fields) or baby (All Fields) or babies (All Fields)	4,359,414	23-Feb-22
#3	sick (All Fields) or illness (All Fields) or disease (All Fields) or diseases (All Fields)	7,257,713	23-Feb-22
#4	quality (All Fields)	3,189,104	23-Feb-22
#5	mother (All Fields) or mothers (All Fields) or care giver (All Fields) or caregivers (All Fields) or care givers (All Fields) or caretaker (All Fields) or care taker (All Fields) or caretakers (All Fields) or care takers (All Fields)	409,692	23-Feb-22
#6	child health care (All Fields) or child healthcare (All Fields) or newborn care (All Fields) or pediatric care (All Fields) or paediatric care (All Fields) or management of childhood illness (All Fields) or integrated management of childhood illness (All Fields) or IMCI (All Fields) or community case management (All Fields) or CCM (All Fields) or integrated community case management (All Fields) or iCCM (All Fields) or community-based newborn care (All Fields) or community based newborn care (All Fields) or CBNC (All Fields)	455,789	23-Feb-22
#7	Ethiopia (All Fields)	58,346	23-Feb-22
#8	#7 AND #6 AND #5 AND #4 AND #3 AND #2 AND #1	63	23-Feb-22

Table 2: Characteristics of the included studies in the systematic review of quality of healthcare provided to sick children in healthcare facilities in Ethiopia, 2022

Author, year	Study design	Study population	Quality component assessed	Type of disease focused	Type of intervention assessed
Abawollo, et al., 2021 [16]	Nonexperimental (cross sectional)	Neonatal intensive care unit (NICUs)	Structure, process, and outcome	Severe newborn illness	Neonatal intensive care
Abayneh, et al., 2020 [17]	Non-randomized (pre-post intervention)	Clinical records of <5 children	Process	Diseases covered in IMCI protocol	Capacity building of HPs and resource supplementation
Akweongo, et al., 2011 [18]	Nonexperimental (cross sectional)	<5 children & their caretakers paired	Structure, process, and outcome	Malaria/fever	Community malaria medicine distribution
Alemnew, 2011 [19]	Nonexperimental (cross sectional)	Children 2 months to 5 years of age	Structure and process	Diseases covered in IMCI protocol	IMNCI protocol implementation
Amouzou, et al., 2016 [20]	RCT	Women aged 15–49 years and children aged 2–59 months	Process and outcome	Malaria, diarrhea, pneumonia, and SAM	iCCM of common childhood illnesses
Awasthi, 2006 [21]	RCT	2-59 months old children with diarrhea	Outcome	Diarrhea	ORS plus Zinc supplementation
Berhane, et al., 2021 [22]	Non-randomized (pre-post intervention)	Infants < 2 months of age with any sign of possible serious bacterial infection (PSBI)	Process and outcome	PSBI	Implementation of WHO's PSBI guideline
Beyene, et al., 2021 [23]	Nonexperimental (cross sectional)	<5 children with severe disease and their caretakers paired	Process	Severe childhood illnesses that require referral service	Using CBNC, iCCM, and IMNCI guidelines for proper referral of children
Biadgo, et al., 2021 [7]	Nonexperimental (cross sectional)	Mothers, newborns and children	Structure and process	MNCHC	MNCHC QI interventions
Daka, et al., 2020 [9]	Nonexperimental (cross sectional)	Children 2-59 months of age who sought care from HEWs at HPs	Outcome	Common childhood illnesses	Optimizing HEP intervention
Defar, et al., 2021 [24]	Qualitative	Mothers of <5 children and HEWs	Outcome	Common childhood illnesses	HEWs based curative care for child
Degefie, et al., 2017 [25]	RCT	Infants <2 months of age with PSBI signs and their caretakers	Structure, process and outcome	PSBI	Trainings of HEWs, volunteers, and HC staff and provision of antibiotics and supplies to HPs and HCs
Funk, et al., 2018 [26]	RCT	HEWs and caretakers of children with unclassified fever	Process and Outcome	Unclassified fever	Management of children by HEWs as per iCCM guideline
Gage, et al., 2018 [27]	Nonexperimental (cross sectional)	Health facilities & service providers who ever managed sick children	Structure and process	Common childhood illnesses	Implementation of IMCI guidelines
Gebremedhin, et al., 2019 [28]	Nonexperimental (cross sectional)	Mothers, HEWs, district health office head and focal persons of PHCUs	Structure, process and outcome	Common childhood illnesses	Implementation of CBNC program
Getachew, et al., 2020 [8]	Nonexperimental (cross sectional)	Caretakers and their sick children	Structure, process and outcome	Common childhood illnesses	Implementation of IMCI guidelines at HPs, HCs, private clinics and hospitals
Hagaman, et al., 2020 [29]	Non-experimental (time series)	HCs and their corresponding HPs	Structure, process and outcome	Neonatal complications (sepsis and asphyxia)	QI clinical skills training with coaching
Jebessa, et al., 2021 [30]	Qualitative	Care providers, health care system administrators and mothers of preterm, LBW and sick newborns	Structure and outcome	Newborn illness	HSS activities, e.g., training of HEWs, volunteers & HC staff & provision of antibiotics & supplies to HPs & HCs.

Kabalo & Seifu, 2017 [31]	Nonexperimental (cross sectional)	Records of children aged 6–59 months with SAM treated at HPs/OTP sites	Process and Outcome	SAM	Implementation of SAM management protocol
Kahsay, et al., 2019 [32]	Nonexperimental (cross sectional)	Young infants aged 0-59 days with PSBI	Process	PSBI	Outpatient management of PSBI by HEWs
Ketaro, et al., 2021 [33]	Nonexperimental (cross sectional)	Caretakers of <5 children who visit HCs for their sick child	Structure, process and outcome	Diseases covered in IMCI protocol	Implementation of IMNCI protocol
Mash, et al., 2003 [34]	Nonexperimental (cross sectional)	Mother-child pairs (age <24 months) and <5 children	Structure	Diarrhea, pneumonia and malaria	Implementation of IMNCI protocol
Mekonnen & Desalegn, 2018 [35]	Nonexperimental (cross sectional)	Selected mother-baby pairs of neonates admitted to NICU	Structure, process and outcome	Severe newborn diseases	Implementation of IMNCI protocol
Miller, et al., 2013 [36]	Nonexperimental (cross sectional)	Children 2–59 months of age	Structure, process and outcome	Illnesses covered in iCCM guideline	Implementation of iCCM program
Najjemba, et al., 2014 [37]	Nonexperimental (cross sectional)	HEWs, <5 children and their caregivers	Structure, process and outcome	Illnesses covered in iCCM guideline	Implementation of iCCM program
Seid, et al., 2021 [38]	Nonexperimental (cross sectional)	Parents who visited the NICU	Outcome	Severe newborn diseases	Service delivery per the national standard of NICU
Shaw, et al., 2015 [39]	Nonexperimental (cross sectional)	Sick <5 children and their caretakers	Outcome	Illnesses covered in iCCM guideline	Implementation of iCCM program
Sintayehu, et al., 2020 [40]	Nonexperimental (cross sectional)	Midwives and nurses providing newborn care	Structure and process	Respiratory distress in newborns	Neonatal resuscitation
Tamiru, et al., 2021 [41]	Nonexperimental (cross sectional)	Caregivers who visited HPs with <5 children for iCCM services and HEWs who provided the services	Process	Diseases covered in iCCM guideline	Implementation of iCCM strategy
Teklu, et al., 2020 [42]	Qualitative	Mothers, service providers and facility administrators	Process	Severe illness of newborns	Functional referral system
Usman, et al., 2019 [43]	Qualitative	Clinical, nursing and administrative leaders	Structure	Illness of newborns	HSS to support PHCUs providing newborn care
Mengistu et al., 2021 [44]	Qualitative	Mothers of <5 children and others	Outcome	Diseases covered in iCCM and CBNC	Implementation of iCCM and CBNC strategies
Tariku, et al., 2022 [45]	Nonexperimental (cross sectional)	Caregivers with children 2–59 months, HPs and HEWs	Structure and outcome	Pneumonia	Optimizing HEP by community engagement, capacity building of HEWs and WDA group leaders, and district health system strengthening
Birhanu and Mathibe-Neke, 2022 [46]	Nonexperimental (cross sectional)	Service providers at HCs and primary hospitals	Structure and outcome	Newborn illness	Implementing IMNCI and iCCM strategies
Tadesse, et al, 2022 [47]	Nonexperimental (cross sectional)	Caretakers of <5 children with diarrhea and their households	Outcome	Diarrhea	Implementing model household strategy of HEP
Kassa, et al, 2022 [48]	Nonexperimental (cross sectional)	Mothers of <5 children	Outcome	Diseases covered in iCCM	Implementing iCCM strategy

RCT: Randomized control trial; NICU: Neonatal intensive care unit; **<***5: under five; HEWs: health extension workers; HPs: health posts; PHCUs: primary healthcare units; HCs: health centers; LBW: low birth weight; SAM: severe acute malnutrition; PSBI: possible severe bacterial infections; iCCM: integrated community case management; IMCI: integrated management of child hood illness; MNCHC: maternal, newborn and child healthcare; CBNC: community-based newborn care; QI: quality improvement; HSS: health systems strengthening; HEP: health extension program; WDA: women's development army*

Table 3: Key findings of the included studies in the systematic review of quality of healthcare provided to sick children in healthcare facilities in Ethiopia, 2022

Author, year	Key findings
Abawollo, et al., 2021 [16]	Low readiness of primary hospitals to deliver NICU services; 63% of hospitals met structural standards; inadequate training; 24%, 65% & 87% of hospitals fulfilled equipment, lab tests & clinical services, respectively; improvement, referral out & death rates at discharge were 71.5%, 18.4% & 6.6%, respectively.
Abayneh, et al., 2020 [17]	Implementation of a complete quality improvement cycle on IMCI interventions significantly improved providers' assessment & classification of childhood illnesses.
Akweongo, et al., 2011 [18]	Community Medicine Distributors (CMDs) prescribed correct dose of Artemisinin-based combination therapies (ACT) for 99% of children with fever; 54% were treated within 24 hours of symptom onset; caretakers' knowledge of malaria signs & symptoms improved post-intervention; caretakers were satisfied with CMDs' services but lacked confidence in CMDs and preferred private facilities for timely diagnosis & treatment.
Alemnew, 2011 [19]	Low quality of IMCI services; no child was completely assessed; long consultation time, lack of support from colleagues & supervisors, inadequate training & drug unavailability were the barriers.
Amouzou, et al., 2016 [20]	Few sick children were taken to iCCM providers. Care seeking increased only marginally in intervention (22.9–25.7%) and comparison (23.3–29.3%) areas. iCCM-trained workers provided good quality care. Mortality declined at similar rates in both arms. Ethiopia's iCCM program did not generate adequate demand & uptake to achieve a meaningful coverage leading to enhanced child mortality reduction.
Awasthi, 2006 [21]	In the management of acute watery diarrhea, zinc plus ORS with culturally appropriate messages in local language did not affect overall ORS use & decreases antibiotic/antidiarrheal use; children had good adherence without side effects.
Berhane, et al., 2021 [22]	Coverage of appropriate treatment for PSBI was 77.7%; 82.2% (n=169) of infants with clinical severe infection were successfully treated as outpatients & 1.3% died. In settings where referral to a hospital is not feasible, young infants with PSBI can be treated on an outpatient basis at a HP or HC, which can contribute to saving many lives.
Beyene, et al., 2021 [23]	Ethiopian PHC providers did not refer nearly half of severely ill children for further care. Parents of referred children often don't comply to the advice. Providers who received in-service training were more likely to adhere to referral guidelines. Factors related to health system, family economy & ambulance services influence whether providers & parents pursue severely ill children's referral.
Biadgo, et al., 2021 [7]	The majority of health facilities did not meet national MNCHC quality standards. Average score was 62% for input, 43% for process & 48% for output domains. Only 5(15.6%), 3(9.3%) & 3(10.7%) of facilities met expected standards for input, process & output domains of quality MNCHC, respectively.
Daka, et al., 2020 [9]	HEWs' clinical assessment, classification & management of sick children did not follow clinical guidelines to a large extent; 53% of sick children were assessed for general danger signs, 89% had their MUAC measured for malnutrition, half received some treatment, & less than one-fifth were referred according to iCCM guidelines. Comprehensive counseling was provided to 38% of caregivers.
Defar, et al., 2021 [24]	Mothers wanted improvements in quality of curative services provided by HEWs to children, while HEWs needed capacity building & more support for providing effective community-based child health services.
Degefie, et al., 2017 [25]	HEWs identified 57% of sick newborns as having at least 1 PSBI sign; 90% of caretakers of infants with PSBI refused referral. Estimated treatment coverage of PSBI at HPs was 50%. Neonatal mortality declined more in the intervention than the comparison arm. When referral is not possible, HEWs can properly provide antibiotic treatment of PSBI in newborns at HP level.
Funk, et al., 2018 [26]	HEWs' understanding of how to handle an unclassified fever diagnosis enabled them to withhold medicines from children with this condition & avoid referral to HCs. Making amendments to guidelines for unclassified febrile illnesses, e.g., including a certain diagnostic term & clearer management instruction, could help HEWs to more confidently manage these cases. While universal follow-up advice provided an opportunity to see the child's health progress, conditional follow-up advice allowed saving time & costs.
Gage, et al., 2018 [27]	Low providers' compliance with an average score of 34%; the know-do gap for assessment was only 1%, while the gap for treatment & counseling items was 39%; Drs had the largest gap between knowledge & performance; provider type & equipment availability significantly predicted the know-do gap.
Gebremedhin, et al., 2019 [28]	Availability of resources was 81% with some essential drugs & equipment out of stock including vitamin K, chlorohexidine ointment, neonatal resuscitation bags & masks. Compliance of providers with national guideline was low (68%). Very severe diseases were not treated according to guidelines & identification of neonatal sepsis cases was poor. Maternal satisfaction with CBNC was low (75%).

Getachew, et al., 2020 [8]	Assessment of sick children was of low quality with many missing procedures. Among children diagnosed with malaria, 51% were assessed for all 3 IMCI main symptoms & 4% were assessed for all 3 general danger signs. 53% of providers received training in the past 2 years; only 33% used IMCI mother's card to remind key messages she received & only 2% of providers used visual aids during consultations. Mean satisfaction score was 76%, caregivers who sought care at HPs reported the lowest mean satisfaction score (71%) compared to those who sought at other facility types. Availability of basic amenities negatively affected satisfaction, while consulting private facilities & training of providers positively affected.
Hagaman, et al., 2020 [29]	Infrastructural capacity improvement following a health systems quality improvement intervention significantly increased providers' adherence to recommended care practices. Perinatal mortality reduced slightly post-intervention.
Jebessa, et al., 2021 [30]	Clinical guidelines for sick newborn care were not available in many facilities. Human resource challenges inhibited care quality. There were widespread shortages of equipment & supplies, medication, physical space, water, electricity & transportation. Clinical guidelines were often not followed. Most providers reported little or no communication with parents following hospital discharge. Caretakers, providers & facility administrators perceived the quality of care was low.
Kabalo & Seifu, 2017 [31]	Recovery rate was lower than the sphere standard. Recovery, death & defaulter rates were 64.9%, 1.2% & 2.2%, respectively. Children living in <25 min & provided with amoxicillin had 1.53- & 1.52-times higher odds of recovery than their counterparts, respectively.
Kahsay, et al., 2019 [32]	Incidence rates of PSBI were 12% & 10% for Raya Alamata & Raya Azebo districts, respectively. 208 PSBI cases needed referral, of which, 69.2% accepted. HEWs can play a significant role in community management of sick young infants & improve newborn health.
Ketaro, et al., 2021 [33]	Almost all providers were not trained on child health or illnesses & no written job description as a work aid for 2 years. Lack of medicine (75%) & poor service quality (65%) were main reasons for not visiting the nearest HC. No provider used a visual aid to educate caretakers, 59.6% of caretakers were informed about the illness, & 48.4% were informed on danger signs & symptoms. Mean satisfaction score with IMNCI service was 63.4(±18.9); 23.4% & 33.6% were dissatisfied with waiting times & explanations about the problem or treatment, respectively. Availability of drugs & lab services significantly affected caretakers' satisfaction.
Mash, et al., 2003 [34]	Most (83%) providers incorrectly prescribed antibiotics & only 50% knew at least 3 referral indications. Caretakers mostly refused referral due to lack of transport or money. About a quarter of caretakers understood treatment recommendations & only 13% were instructed on follow-up cares.
Mekonnen & Desalegn, 2018 [35]	Equipment & drugs were not fulfilled in the NICU ward. Only 65.2% of equipment, 72.2% of medicines & 81% of lab tests were fulfilled. Blood chemistry & culture & sensitivity tests of any fluid were not available.
Miller, et al., 2013 [36]	Nearly all HEWs were trained on iCCM & 87% received supervision in the previous 3 months. 70% of HPs had all essential commodities for iCCM; HEWs in iCCM intervention areas performed most basic assessment tasks & correctly managed nearly two-thirds of all children, with minimal over-prescription of drugs. Fewer children (62%) were assessed for all 4 general danger signs & 53% were correctly classified for all major iCCM illnesses. 72%, 79% & 59% were correctly managed for pneumonia, diarrhea & malnutrition, respectively. HEWs correctly managed only 34% with severe illness & referred about half needing referral.
Najjemba, et al., 2014 [37]	Over 80% of the HPs had essential drugs for sick children on the day of assessment; 13% did not have plumpy Nut Sachets; RDTs & timers were available in 85% of the HPs; disposable gloves & Ambu bags were not available in most HPs; HEWs' knowledge on assessments & classification & referral indications was >80%. Quality of care provided by HEWs & caregivers' compliance to prescribed medicines were good. But treatment follow-up, provision of recommended dosage for age & identifying danger signs needed improvement.
Seid, et al., 2021 [38]	Half of the parents were dissatisfied with care given to their neonates during their hospital stay. Parents agreed with availability of restroom around waiting area (26%) & visiting hours are flexible (41%). Parents disagreed with child's room is quiet enough (25%) & doctor's use of medical terms explaining what they mean during the visit to NICU (26%). Length of stay was significantly associated with parental satisfaction (AOR = 2.42).
Shaw, et al., 2015 [39]	Utilization of iCCM services was low with only 9.3% of caregivers of a child with diarrhea, fever, &/or pneumonia in 2 weeks seeking care from HEWs. Perceptions that HP was not open (20.7%), drugs were not available at the HP (18.7%) & poor service by HEWs or at the HP (15.0%) were the barriers.
Sintayehu, et al., 2020 [40]	Most health facilities had neonatal resuscitation corners (97.4%), adequate newborn resuscitation guidelines (63%), & essential equipment (85%). However, only 25.1% professionals ever received refresher training on neonatal resuscitation & 47.3% ever performed the procedure. Basic neonatal resuscitation skills of midwives & nurses in Eastern Ethiopia were not well retained, at a rate of 11.2%.
Tamiru, et al., 2021 [41]	More than half of HEWs correctly assessed, classified & prescribed drugs for ICCM cases.
Teklu, et al., 2020 [42]	Lack of ambulance was among the most common barriers to newborn referral system in both urban & rural settings.

Usman, et al., 2019 [43]	Adequate space, power & water were problematic in the public health system. Human resource issues were critical to properly care for sick newborns. Widespread problems exist with functional equipment & equipment distribution systems. Facilities lacked funds to support sick newborn needs. Key facilitators, information disseminators & influencers were HDA, community & religious leaders & mothers & families who had positive experiences or outcomes of care.
Mengistu et al., 2021 [44]	HP closure & drug stock-out led to inconsistent service availability. Limited confidence & skill of HEWs & under-resourced physical facilities affected service delivery.
Tariku, et al., 2022 [45]	Only 62.8% of the HPs were ready to provide sick childcare & 85.1% were functionally open for >5 days per week. Most HEWs (78.8%) were trained on iCCM & 78.8% received supportive supervision in 6 months. Mean availability of essential drugs was 66.9%, half of the HPs had RDT for malaria & very few HPs had all essential drugs. Utilization of curative services for childhood suspected pneumonia at HPs was low.
Birhanu and Mathibe-Neke, 2022 [46]	Availability of essential equipment was associated with quality of neonatal care in health facilities ($p < 0.05$). Effectiveness of neonatal healthcare services was associated with health facility readiness score.
Tadesse, et al, 2022 [47]	Model household strategy of HEP contributed a 17.7% decrease in childhood diarrhea. Children from non-model households were 2.19 times more likely to develop diarrheal diseases than their counterparts. Households which got no, or infrequent home visits were 3.28 times more likely to develop childhood diarrhea.
Kassa, et al, 2022 [48]	Low iCCM uptake (47.2%); awareness of iCCM service (AOR = 3.73) & training on HEP packages as model family (AOR = 2.07) significantly improved the uptake.

NICU: Neonatal intensive care unit; iCCM: integrated community case management; PSBI: possible severe bacterial infections; HPs: health posts; HCs: health centers; PHC: primary healthcare; MNCHC: maternal, newborn and child healthcare; HEWs: health extension workers; MUAC: Mid-upper arm circumference; CBNC: community-based newborn care; IMCI: integrated management of childhood illness; RDT: Rapid diagnostic test; HEP: health extension program
